# Circadian Variation in the Onset of Placental Abruption

**DOI:** 10.1155/2017/3194814

**Published:** 2017-01-09

**Authors:** Masanao Ohhashi, Seishi Furukawa, Hiroshi Sameshima

**Affiliations:** ^1^Department of Obstetrics & Gynecology, Faculty of Medicine, University of Miyazaki, Miyazaki, Japan; ^2^Department of Obstetrics & Gynecology, School of Medicine, Kyorin University, Tokyo, Japan

## Abstract

*Objective*. To determine circadian variation in the onset of placental abruption.* Methods*. A retrospective study involving 115 placental abruptions, divided into four subgroups based on initial symptoms comprising abdominal pain, vaginal bleeding, both abdominal pain and bleeding, or other symptoms. The time of the initial symptom was considered the disease onset. We analyzed the frequency of disease onset and adverse perinatal outcome including perinatal death relative to the daily four 6-hour intervals.* Results*. Abdominal pain displayed significant circadian variation regarding the period of onset with higher levels from 0:00 AM to 6:00 AM (65%) compared with 0:00 PM to 6:00 PM (24%, *p* < 0.01). Vaginal bleeding did not display significant circadian variation (*p* = 0.45). Adverse perinatal outcome showed significant circadian variation with a higher occurrence of perinatal death from 0:00 AM to 6:00 AM (35%) compared with 0:00 PM to 6:00 PM (0%, *p* < 0.01). After adjustment using variables of abdominal pain and time period, both variables significantly affected perinatal death (odds ratio: 13.0 and 2.2, resp.). The risk of adverse perinatal outcome increased significantly when abdominal pain occurred, except for the period 0:00 PM to 6:00 PM (OR, 9.5).* Conclusion*. Placental abruption beginning with abdominal pain has circadian variation.

## 1. Introduction

Perinatal asphyxia is one of the major risk factors for poor prognosis, including perinatal death and brain damage [1]. Among cases of perinatal asphyxia, placental abruption accounts for 58% of perinatal deaths and 26% of cases involving brain damage [1]. It has been difficult to predict the onset of placental abruption, and the diagnosis of placental abruption is always clinical. In order to improve perinatal outcomes, the prediction of disease severity based on clinical features is more practical. There are currently several reports detailing the prediction of disease severity based on clinical features. For example, fetal heart rate pattern and clinical manifestation such as preterm PROM or preeclampsia are closely related to perinatal outcomes [2, 3]. However, the relationship between the timing of disease onset (i.e., circadian variation in the onset of placental abruption) as a clinical feature and disease severity is not fully understood. In particular, the presence of circadian variation in the onset of placental abruption is not obvious.

Ischemic events, including myocardial infarction or stroke, have shown a certain circadian rhythm regarding onset of disease, and reports indicate that the onset of diseases frequently occurs between 6 AM and noon [4, 5]. The discrepancy between blood supply and demand before noon may be responsible for the high occurrence of ischemic events [6]. There are presently two major etiologies that have been suggested for the development of placental abruption. One is an inflammation-associated process and the other is an ischemia-associated process [7]. According to our previous study, ischemic changes of the placenta were common in a group involving placental abruption with hypertension, and infarctions of the placenta were common in the group involving placental abruption with low-risk pregnancy compared to the group with placental abruption and a risk of premature labor or preterm PROM [3]. These findings indicate that ischemic events participate at certain stages of the pathogenesis of placental abruption, and placental abruption with ischemic changes may show daily fluctuation regarding disease onset. Therefore, we conducted the study to determine circadian variation in the onset of placental abruption and associated perinatal outcomes.

## 2. Materials and Methods

The current study was conducted in the setting of two perinatal centers. We retrospectively examined the medical charts of women with placental abruption that were admitted to the Perinatal Center of the University of Miyazaki or the Perinatal Center of the Miyazaki Medical Association Hospital from January 1997 to April 2013. The Perinatal Center of the University of Miyazaki is a tertiary center, whereas the Perinatal Center of the Miyazaki Medical Association Hospital is a secondary perinatal center. In our area, 80% of pregnant women give birth at a private clinic. Both centers dealt primarily with referral cases from private clinics, and the total number of deliveries was 11249 during the study period. These two centers can perform an emergency cesarean section within 30 minutes if detecting NRFHRs necessitating the termination of pregnancy. A neonatal specialist is present at delivery. An anesthesiologist is present at the time of a cesarean section.

We utilized the definition of placental abruption used in previous studies [2, 3, 8]. Briefly, placental abruption was determined by the presence of retroplacental hematoma at delivery and clinical manifestations (any one or combination of vaginal bleeding, abdominal pain, pregnancy-induced hypertension, premature labor, premature rupture of membrane, intrauterine fetal death, or a nonreassuring fetal heart rate pattern).

During the investigation period, we identified 131 cases of placental abruption. We examined self-reported documents of symptoms before admission for all cases. We excluded from the study cases with multifetal pregnancies and without self-reported documents of symptoms. Finally, a total of 115 pregnancies displaying placental abruption were enrolled in this study. The following maternal and neonatal characteristics were collected: maternal age, parity (primipara), gestational age at delivery (weeks), smoking (>1 packet per day), birth weight (g), cesarean delivery, hypertension in pregnancy including chronic hypertension, preeclampsia, gestational hypertension, premature rupture of the membranes (PROM), and prior history of placental abruption. Proteinuria without hypertension was excluded from the group of hypertension in pregnancy. Adverse perinatal outcomes including perinatal death (intrauterine fetal death: IUFD and neonatal death: ND), low umbilical artery pH (UA pH < 7.0), and low Apgar score at 5 minutes (<7) were investigated.

We examined initial symptoms recorded from an interview on or after admission. The 115 cases of placental abruption were divided into four subgroups according to initial symptoms. Four initial symptoms included abdominal pain, vaginal bleeding, both abdominal pain and bleeding occurring simultaneously, or other symptoms. When abdominal pain was preceded by bleeding, we classified the case into the abdominal-pain group. When bleeding preceded abdominal pain, we classified the case into the vaginal-bleeding group. In this study, we defined the time of the initial symptom as the onset of placental abruption. Other symptoms included any conditions unaccompanied by abdominal pain, vaginal bleeding, or both.

We analyzed the frequency of disease onset based on the initial symptom for the four 6-hour intervals in a day: 0:00 AM to 6:00 AM, 6:00 AM to 0:00 PM, 0:00 PM to 6:00 PM, and 6:00 PM to 0:00 AM. Adverse perinatal outcomes were also analyzed relative to the four 6-hour intervals. Comparisons for maternal and neonatal characteristics among groups were made using the one-way ANOVA or *χ*^2^. Differences in disease onset and perinatal outcome for the four 6-hour intervals were analyzed using *χ*^2^ tests and Steel-Dwass multiple comparison tests. Univariate and multiple logistic analyses were then performed to determine the effect of circadian variation on poor outcome. Data are expressed as number, incidence (%), mean ± SD, or range. Probability values < 0.05 were considered significant.

We obtained approval (#2016-2) from a constituted Ethics Committee at our institution.

## 3. Results

The 115 cases of placental abruptions were divided into four groups comprising abdominal pain (*n* = 53), vaginal bleeding (*n* = 35), abdominal pain and bleeding (*n* = 3), or other symptoms (*n* = 24) according to the initial symptom. Other symptoms are listed in Table 1.

The frequency of disease onset based on any initial symptom was 35% (*n* = 40) for the period 0:00 AM to 6:00 AM, 23% (*n* = 26) for 6:00 AM to 0:00 PM, 22% (*n* = 25) for 0:00 PM to 6:00 PM, and 21% (*n* = 24) for 6:00 PM to 0:00 AM. There was no significant difference in maternal characteristics as represented by maternal age, parity, gestational age at delivery, smoking, hypertension, PROM, prior history of placental abruption, or the incidence of cesarean delivery among groups for the four 6-hour intervals (Table 2). Additionally, there was no significant difference in birth weight among groups for the four 6-hour intervals (*p* = 0.16, Table 2).

**Figure 1:**
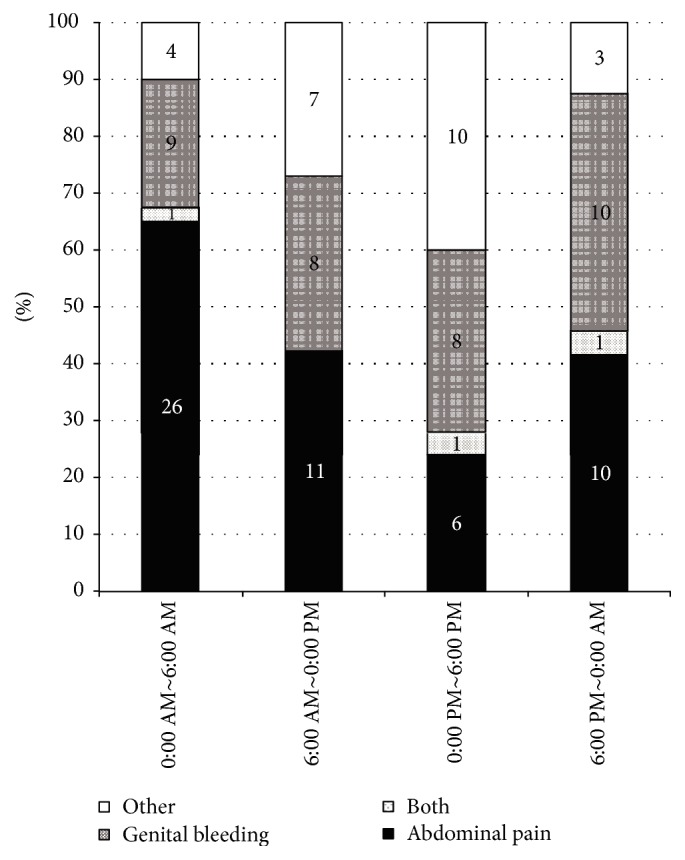
Circadian pattern of onset of disease based on initial symptoms. Initial symptoms included abdominal pain (white), vaginal bleeding (striped), abdominal pain and bleeding (dark), or others (gray). Comparisons of incidence of symptom-oriented disease onset among groups for the four 6-hour intervals in regard to placental abruption were made using *χ*^2^ tests followed by the Steel-Dwass test for multiple comparisons. A higher level of abdominal pain from 0:00 AM to 6:00 AM was detected when compared with 0:00 PM to 6:00 PM (*p* < 0.01).

Abdominal pain as an initial symptom displayed significant circadian variation with values of 65% for 0:00 AM to 6:00 AM, 42% for 6:00 AM to 0:00 PM, 24% for 0:00 PM to 6:00 PM, and 42% for 6:00 PM to 0:00 AM (*p* < 0.01, Figure 1). In particular, a higher level of abdominal pain from 0:00 AM to 6:00 AM was detected when compared with 0:00 PM to 6:00 PM (*p* < 0.01). In contrast, vaginal bleeding did not show circadian variation with values of 23% for 0:00 AM to 6:00 AM, 31% for 6:00 AM to 0:00 PM, 32% for 0:00 PM to 6:00 PM, and 42% for 6:00 PM to 0:00 AM (*p* = 0.45).

**Figure 2:**
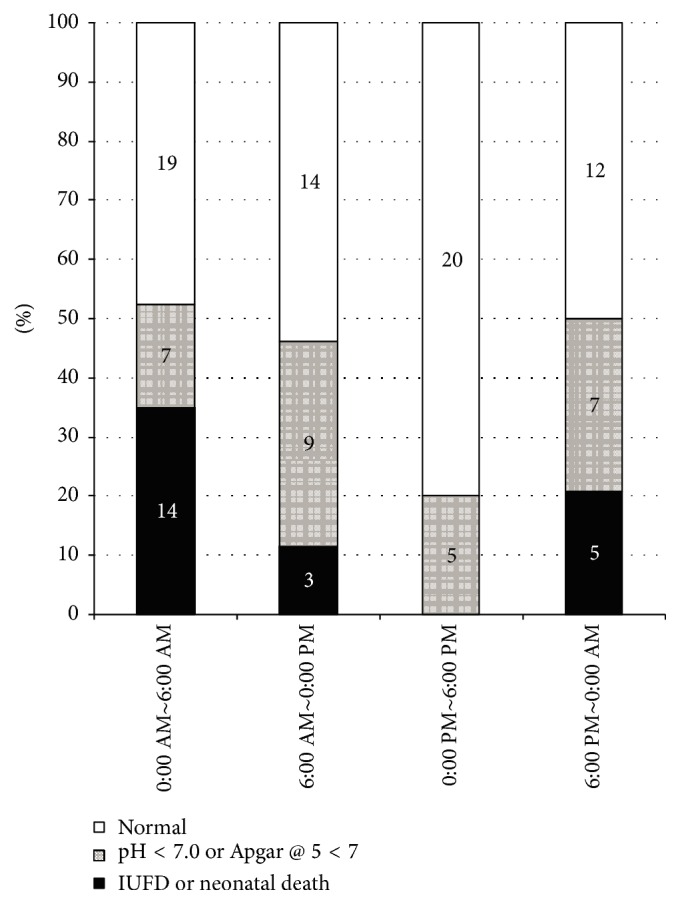
Circadian pattern of severe perinatal mortality. A case having IUFD, ND, low umbilical artery pH (UA pH < 7), or a low Apgar score at 5 minutes (<7) was regarded as having a severe perinatal outcome. Dark bar shows IUFD or ND. Striped bar represents low UA pH or a low Apgar score. White bar corresponds to the absence of severe perinatal mortality. Comparisons of the incidence of severe perinatal mortality among groups for the four 6-hour intervals in regard to placental abruption were made using *χ*^2^ tests followed by the Steel-Dwass test for multiple comparisons. A higher occurrence from 0:00 AM to 6:00 AM was detected when compared with 0:00 PM to 6:00 PM (*p* < 0.05). IUFD: intrauterine fetal death. ND: neonatal death.

Adverse perinatal outcome showed significant circadian variation with values of 53% for 0:00 AM to 6:00 AM, 46% for 6:00 AM to 0:00 PM, 20% for 0:00 PM to 6:00 PM, and 50% for 6:00 PM to 0:00 AM (*p* = 0.01, Figure 2). In particular, a higher occurrence from 0:00 AM to 6:00 AM was detected when compared with 0:00 PM to 6:00 PM (*p* < 0.05). Analysis of the occurrence of perinatal death revealed a higher incidence (twelve cases of IUFD and two cases of neonatal death) from 0:00 AM to 6:00 AM when compared with the period 0:00 PM to 6:00 PM, during which there were no cases of perinatal death (*p* < 0.01, Figure 2). Our analysis also revealed three cases of IUFD for the period 6:00 AM to 0:00 PM and five cases of IUFD for the period 6:00 PM to 0:00 AM.

As abdominal pain and adverse perinatal outcome displayed significant circadian variation, we performed multiple logistic analysis using the variables of abdominal pain and time period. For this purpose, we allocated women with abdominal pain a score of 1 and those without abdominal pain a score of 0. Since a stepwise difference of perinatal death was noticed along with time period, we allocated the four time periods the following scores: 3 (0:00 AM to 6:00 AM), 1 (6:00 AM to 0:00 PM), 0 (0:00 PM to 6:00 PM), and 2 (6:00 PM to 0:00 AM). After adjusting two cofounding factors, we found that abdominal pain (adjusted OR 13.0, 95% CI 2.8–60.9) and time period (adjusted OR 2.2, 95% CI 1.2–4.0) were independent factors associated with perinatal death. We also performed univariate analysis. For this purpose, the 115 cases of placental abruptions were divided into two groups: women experiencing abdominal pain at 0:00 AM to 6:00 AM, 6:00 AM to 0:00 PM, or 6:00 PM to 0:00 AM and others. The risk of adverse perinatal outcome increased significantly when placental abruption with abdominal pain occurred during a period other than 0:00 PM to 6:00 PM (*p* < 0.01, odds ratio 9.5, and 95% CI 4.0–22.5).

## 4. Discussion

We have shown that placental abruption beginning with abdominal pain exhibited significant circadian variation. On the other hand, placental abruption beginning with vaginal bleeding did not show circadian variation. Perinatal death also exhibited significant circadian variation. The rate of adverse perinatal outcome rose by up to 9.5 times when placental abruption with abdominal pain occurred during a period other than 0:00 PM to 6:00 PM. It is currently difficult to predict the onset of placental abruption. Furthermore, the disease often advances rapidly toward a serious condition. The prediction of disease severity based on clinical features is then more practical in order to improve perinatal outcomes. Thus, the identification in this study of a time period with a higher incidence of disease onset based on the initial symptom and higher risk of poor perinatal outcome is extremely useful as an adjunctive indicator of therapeutic intervention for placental abruption.

A study of circadian variation of disease onset revealed seasonal variation for placental abruption [9]. That investigation showed that placental abruption occurred more frequently in spring and autumn and was associated with unstable weather. It has also been reported that the incidence of preterm PROM is influenced by unstable weather especially in spring and autumn and that women with preterm PROM have a higher risk of developing placental abruption [10, 11]. Thus, the existence of seasonal circadian variation in placental abruption may be confirmed, but the existence of diurnal circadian variation of disease onset remains unclear. Luque-Fernandez et al. investigated placental abruptions and reported a diurnal circadian rhythm with a morning peak at around 4:00 AM, although their results showed no statistical significance [12]. They also reported a significant diurnal circadian pattern with a morning peak at 7:32 AM in moderate preterm PROM cases [12]. In our study, placental abruption beginning with abdominal pain displayed significant circadian variation regarding the time of onset, with a higher incidence recorded during 0:00 AM to 6:00 AM. However, there were no significant differences in the incidence of hypertension and PROM among the four subgroups between the four 6-hour intervals. Accordingly, we did not detect any causal relationship or circadian rhythms between risk factors for placental abruption and disease onset for the four 6-hour intervals in a day. However, investigation of the incidence of hypertension in groups with abdominal pain or bleeding showed a higher tendency for hypertension in the abdominal-pain group (26%) compared to the bleeding group (11%, *p* = 0.09, *χ*^2^ test). According to our previous study, ischemic changes of the placenta were common in a group involving placental abruption with hypertension [3]. Ischemic changes might therefore play a role in the onset of placental abruption beginning with abdominal pain.

Several types of ischemic events such as myocardial infarction display circadian variation in which the onset is more frequent between 6 AM and noon [4, 5]. Blood pressure increases in the morning and is followed by increasing energy and oxygen demand. The vascular tone of the coronary artery also increases during this period. Consequently, the difference between blood demand and supply provokes cardiac disease [6]. During pregnancy, the blood supply of the uteroplacental unit increases along with gestation [13]. In addition, vascular tone during the morning is higher than that during the afternoon [14], and blood pressure during the daytime is higher than that at nighttime during pregnancy [15]. In our study, the frequency of disease onset during the AM period was higher than that of the PM period. The difference between blood demand and supply during the morning may also provoke placental abruption. In fact, infarctions of the placenta are frequently seen in placental abruption [3]. Furthermore, a decrease of the day-night blood pressure difference has been reported in preeclampsia [16]. If lacking a decrease of vascular tone at night, pregnant woman may be more susceptible to the onset of disease during midnight to early morning (0:00 AM to 6:00 AM).

The present study has two limitations. First, we defined the time of initial symptom as the onset of placental abruption and then analyzed the frequency of disease onset based on the initial symptom for the four 6-hour intervals in a day. Since data was derived from an interview on or after admission, there may be a bias regarding the symptom recognized and its onset. We know there was no reliable marker for early recognition of placental abruption, and therefore the onset and diagnosis of disease were only made on the basis of clinical manifestation. The second limitation of this study is the small sample size of the study population. We need a greater sample size in order to better investigate the causal relationship or circadian rhythms between risk factors for placental abruption and disease onset for the four 6-hour intervals in a day.

In conclusion, placental abruption beginning with abdominal pain has circadian variation in that there is a higher incidence of placental abruption during the period 0:00 PM to 6:00 PM. Prognosis becomes worst when placental abruption beginning with abdominal pain develops during the period of 0:00 AM to 6:00 AM. Our results provide additional important information regarding clinical features related to disease severity, as well as useful information to further elucidate the pathogenesis for developing to placental abruption.

## Figures and Tables

**Table 1:** Other symptoms included any conditions unaccompanied by abdominal pain, vaginal bleeding, or both.

Condition	*n* = 24
Threatened premature labor	10
Premature rupture of membrane	6
Nonreassuring fetal status	6
Loss of fetal movement	1
Oligohydramnios	1

Data are expressed as number.

**Table 2:** Demographic data of each group for the four 6-hour intervals in regard to placental abruption.

	0:00 AM ~ 6:00 AM	6:00 AM ~ 0:00 PM	0:00 PM ~ 6:00 PM	6:00 PM ~ 0:00 AM
Number	40	26	25	24
Age (yrs.)	29.1 ± 5.0	30.2 ± 5.7	28.7 ± 4.8	30.3 ± 4.3
Nulliparity (%)	21 (53%)	9 (35%)	12 (48%)	7 (29%)
Smoking	2	2	0	1
Hypertension	9	7	2	3
PROM	2	4	4	0
PPA	2	0	1	0
Gestational age at delivery (wks.)	34.2 ± 4.0	32.0 ± 4.6	33.0 ± 3.5	34.0 ± 4.1
Cesarean delivery	32 (80%)	22 (85%)	20 (80%)	18 (75%)
(IUFD followed by cesarean delivery)	(7)	(1)	(0)	(0)
Birth weight (g)	2135 ± 671	1820 ± 802	1831 ± 536	2123 ± 796

Results are expressed as number, mean ± SD, or incidence (%). PROM: premature rupture of the membranes. PPA: prior history of placental abruption. IUFD: intrauterine fetal death. CD: cesarean delivery.
